# Coping with dry eyes: a qualitative approach

**DOI:** 10.1186/s12886-018-0671-z

**Published:** 2018-01-16

**Authors:** Sharon Yeo, Louis Tong

**Affiliations:** 1 Singapore National Eye Centre, Head, Ocular Surface Research Group, Singapore Eye Research Institute, 11 Third Hospital Avenue, Singapore, 168751 Singapore; 2 Cornea and External Eye Disease Service, Singapore National Eye Center, Singapore, Singapore; 3 Eye Academic Clinical Program, Duke-NUS Medical School, Singapore, Singapore; 4 Department of Ophthalmology, Yong Loo Lin School of Medicine, National University of Singapore, Singapore, Singapore; 5 Singapore Eye Research Institute, Ocular Surface Research Group, 11 Third Hospital Avenue #05-00, Singapore, 168751 Singapore

**Keywords:** Dry eye, Focus group, Qualitative methods, Holistic care, Human disease, Ocular surface disease, Keratoconjunctivitis sicca

## Abstract

**Background:**

Dry eye is a common problem that affects many people worldwide, reducing quality of life and impacting daily activities. A qualitative approach often used in medicine and other disciplines is used to evaluate how people with dry eye cope with this impact.

**Methods:**

Six focus group sessions were conducted at the Singapore National Eye Centre (SNEC), premises of an eye research institute. These focus groups consist of a spectrum of dry eye sufferers (30 women, 8 men, aged 61 ± 11.8 years). Standard methods of coding followed by determination of themes were adhered to. Where classification was difficult, consensus was made between 3 assessors.

**Results:**

Audio-recorded transcripts were coded in 10 themes by 3 assessors independently. Four of the themes involved traditional measures such as lid warming, cleansing, lubrication and oral dietary supplements. The other themes discovered were Traditional Chinese Medicine, modification of eye-care habits (e.g. wearing sunglasses), environmental humidity, lifestyle (e.g. sleeping habits), psychological attitude, and lastly sharing and communication.

**Conclusion:**

Holistic coping strategies were found to be prominent in dry eye sufferers from these focus groups, and people tend to find personalised ways of coping with the impact of dry eye on daily living.

**Electronic supplementary material:**

The online version of this article (10.1186/s12886-018-0671-z) contains supplementary material, which is available to authorized users.

## Background

Dry eye disease (DED) is a common cause of distress in patients that is often overlooked [1]. The International Dry Eye Workshop II defined DED as a “a multifactorial disease of the ocular surface characterized by a loss of homeostasis of the tear film, and accompanied by ocular symptoms, in which tear film instability and hyperosmolarity, ocular surface inflammation and damage, and neurosensory abnormalities play etiological roles” [2].

In this article, we explore the role of various patient activity-initiated, cultural and lifestyle factors in the management of dry eye. This follows previous findings that showed patients having a tendency to adjust activities of daily living to cope with dry eyes [3]. It has also been found that symptoms of dry eye was only weakly associated to decrease in quality of life, this implied that quality of life is not directly due to the symptoms of dry eye but could be more related to the success of patient’s lifestyle and their strategies to cope with dry eye [4]. It is essential to study the various self-management methods in a condition that is chronic, episodic and non-curable. Therefore we aim to explore these self-management factors using a qualitative approach, and the findings will have implications on future dry eye management and how we assess treatment outcomes to improve patient’s quality of life. This approach is novel in the field of dry eye.

### Literature review

Dry eye is a condition of diverse aetiology that is estimated to affect 7.4% to 33.7% of the population [5, 6]. DED has been found to cause a considerably negative impact on the quality of life (QoL) [7–9], and imposes a significant socio-economic burden [10, 11]. As a result, the QoL of patients, including aspects of physical, social, psychological functioning, daily actions and workplace productivity, is greatly affected [12].

One of the reasons why dry eye is so debilitating is the near ubiquitous nature of its risk factors.

Known risk factors for DED includes aging [13], female sex [14], hormonal changes [15, 16], (e.g., post-menopausal) [17], eyelid disease [1], refractive surgery [18], autoimmune disease [19], and smoking [20]. Environmental risk factors for DED includes wearing of contact lenses [1], low humidity [21] (e.g., air-conditioned environment), exposure to sun, dust and wind [22], medications [23]—topical eye-drops with preservatives and systemic medications like antihistamines and antidepressants—and extended visual tasking [24, 25] that includes computer use, watching of television, prolonged reading, and driving.

Currently, symptomatic relief for dry eye includes the use of emulsions, gels, ointment or tear substitutes to lubricate the ocular surface. Alternatively, studies have shown that good eye lid hygiene through lid cleaning, as well as lid warming encourages the production of tears and therefore reduce the discomfort caused by dry eyes. Apart from medical treatment, lifestyle changes have also been effective for some patients. Humidifying ambient air, avoiding air drafts and changing the characteristic of airflow at work, at home and in the car may be helpful [3]. Indeed, the urban office environment has been implicated as the major cause of the ‘computer vision syndrome’, a variant of dry eye [3, 21].

Traditional therapies such as Traditional Chinese Medicine (TCM) in the form of acupuncture or herbs have been evaluated in the findings of the TCM randomised controlled trials [26]. In Singapore, a significant proportion of registered institutional TCM practitioners showed an interest in the management of dry eye [27]. Herbal and acupuncture treatment in TCM have been studied in a scientifically valid manner showing that acupuncture was effective in increasing tear secretion in patients with DED [28]. Meta-analyses also shows evidence that acupuncture therapy is more effective than tears lubricants in terms of tear break up time, Schirmer’s test and corneal fluorescein staining in DED [29, 30].

There are various factors that cause and exacerbate the symptoms of dry eye. A study has also shown association between happiness and DED, suggesting the presence of psychosocial factors in dry eye [31]. Certain systemic diseases affect tear production, and the use of systemic medications and eye surgeries can aggravate dry eye [13]. More importantly, prolonged visual activities, has been shown to stress the ocular surface [3]. For example, we showed that increased duration of television watching was significantly correlated to increased blurring episodes [32]. On the other hand, the exposure of some aggravating factors was clearly manifested, as the hours spent in driving, reading, and exposure to windy environments were significantly correlated to the frequency of dry eye symptoms [32].

A previous author has described 6 psychological types of dry eye patients [33]. For example, one such type will be patients who crave for ‘All Natural Therapy’, believing in herbs and traditional therapies but not drugs with immunosuppressive effects. Another type will be ‘tried it all’ patient who do not believe there is anything a physician can do for them. There is therefore a complex interplay of social and cultural factors (attitudes and beliefs instead) that determine what is acceptable to every dry eye patient individually. There has been no study of social activities in dry eye patients in an Asian context. Previously, we showed that physical activities, such as driving at night, and recognising friends, climbing stairs, etc. were particularly impaired in Singapore Malays in a population based study [32] and these limitations affect their ability to socialise. However, it is unclear if findings in Malays can be extrapolated to the general multi-ethnic population.

There are complex psychosocial profiles in dry eye, which represents interplay between the type of symptoms of dry eye, triggering factors for the symptoms, social and occupational activities of patients, the personally preferred and culturally acceptable treatment and the effectiveness to ease the symptoms experienced. However, to date, there has yet been a holistic study conducted to explore these factors in dry eye patients. We aim to use a focus group based qualitative analysis method to better understand dry eye patients and their preferential coping strategies based on their circumstances and niches.

## Methods

### Methodological approach

Focus groups were chosen as the means of data collection for these reasons: Firstly, interaction between participants encourages ideas and opinions to be shared freely. Secondly, focus group gives participant a chance to consider the subject in greater breadth than they would have previously; also, it encourages participation from individuals who are unwilling to be interviewed on their own. This method is useful in exploring knowledge and experiences of people, as well as to fill in the gaps between health knowledge and patient’s behaviour [34].

Six focus group sessions were conducted at the Singapore National Eye Centre (SNEC), the number of focus groups was not pre-determined but was continued vuntil theme saturation, as recommended in the literature [35]. Each focus group session consists of 4 to 9 participants, meeting the suggested recommended size [34]. The study was approved by the Institutional Review Board and written informed consent was obtained from all participants. Most of the patients were recruited from the dry eye clinic at SNEC, which is a tertiary referral hospital in Singapore, but the clinic is not limited to physician-referred patients. Participants could be private, self-referred patients at SNEC, and the focus group participants were also recruited from the clinical trial centre in Singapore Eye Research Institute. Patients treated with different conventional medicine were recruited.

#### Eligibility criteria

English speaking participants having dry eyes symptoms who are willing to share were recruited in these focus groups. They must also be free of significant hearing loss, cognitive impairment, and must be capable of giving informed consent. This initial screening has been performed by physician or optometrist who normally services the clinic and has been satisfied that the diagnosis of chronic dry eye could apply.

#### Procedure

Each focus group lasted from 1 to 2 h and the sequence adhered to standard guidelines for focus groups described in literature [36]. Pilot testing was performed during the routine clinics. The facilitator, an optometrist, not the ophthalmologist servicing the dry eye clinic was appointed. She has received instructions from training courses on qualitative research, and the same facilitator conducted all 6 focus groups. The topic to be investigated would be described by the facilitator and subsequently the participants introduced themselves. Prior to these sessions, participants agreed that they would be audio-recorded and the recordings would be transcribed for analysis. The self-introduction was helpful to create an association between the participants’ voices and their identity. The sessions were held at a quiet training area away from routine clinical activities in the SNEC building, on mornings when no clinics were conducted.

The introduction phase was followed by individual sharing of each participant on the topic in a consecutive way to ensure equal participation from all who attended. A standardised interview script was used in all the focus groups to guide participants in their sharing (See Additional file 1). Participants were permitted and also encouraged to discuss points raised by others. All participants were given an opportunity to voice their own views in addition to sharing on points brought up by others. At the end of the sessions, the main issues raised and opinions made were summarised by the facilitator. The facilitator had not introduced her own views, as far as possible, concerning the topic or the opinions from the participants, and she ensured that the sessions had not been conducted in a manner that implied her scientific or medical authority in the field of dry eye. The participants were allowed to comment on the verbal summary at the end of the sessions but were not given an opportunity to review the focus group transcripts.

Refreshments provided in the middle of these sessions were sponsored voluntarily by various commercial entities (Bausch and Lomb, LF Asia, Advanced healthcare) who were familiar with various academic and clinical activities of the research group in dry eye. Sponsors did not have any role in the study design or the data analyses arising from the focus groups. They did not participate directly or make any presentations during the focus group. During the introduction phase of the focus groups, participants had been told that the investigators do not formally endorse any products from these sponsoring companies.

### Data analysis

With the exception of occasional instances where more than one voice was heard simultaneously, the recording was clear enough to be transcribed. Each focus group was verbatim transcribed and analyzed. In the event of non-grammatical statements, we retained the original phrases and sentences. In general there was no difficulty identifying specific participants based on distinctive features of the voice, so a unique ID was assigned to each participant. Two optometrists and one ophthalmologist (“the investigators”) involved in the clinical care of dry eye patients scrutinised the transcript. After reading the transcript, a list of codes was created (codebook) to capture the different strategies that were mentioned during the focus groups that the participants had adopted in their daily life to cope the problems arising from dry eye. These were the actual phrases used by participants. This codebook was created to ensure reproducible results and was discussed between investigators before it was finalised. For subsequent focus group sessions, the same codebook was used to code the resulting transcripts. New codes were added to the codebook and further developed as the focus groups were in progress.

The “insert comment” function of Microsoft word software was used in the textual analysis. Content analysis was used in the identification of core meanings or themes in the transcribed text. Such content analysis was used to identify emerging themes in the transcribed text, according to the method of thematic analysis used by *Braun* and *Clarke* [37]. This approach has been shown to be useful for analysis of qualitative data, in contexts outside dry eye, especially for the purpose of identifying core themes and their meaning within the transcript [38].

Consensus on the concepts, categories and themes was determined between the investigators. In cases where some points raised by participants had been either hard to classify or could be classified under more than one category, the investigators had discussed those issues and made a consensus. In order to come to a consensus, the strategy used by the participant will be evaluated in the context where it is employed to determine the coded theme.

Focus groups were scheduled and conducted until theme saturation was achieved, ie., no further themes or categories emerging after a new focus group session [39]. In qualitative approaches, saturation implied that it would not be necessary to continue more sessions or participants as they would be unlikely to introduce novel concepts or ideas [40, 41].

### Profile of participants

Data were collected from 38 individuals, with age ranging from 26 to 85 years old, with a mean age of 61 years (SD: 11.8) over the six focus groups. There was a majority of women (30 women, 8 men) which closely resembles the gender ratio in the dry eye clinic [42]. In populations reported elsewhere, there were also female preponderance but to a lesser extent [43–45].

All except 3 participants were of Chinese ethnicity. Most female participants except 3 were post-menopausal. The patients come from different districts of Singapore, with no predominant estate or locality. Based on their housing type, the participants were from a wide spectrum of social economic background. Although 80% of Singapore’s population lived in subsidized housing, these could be residences of 3, 4 or 5 rooms. There was a balanced mixture of people who were retired from formal occupation as well as busy professionals. There was no deliberate effort to keep each focus group session homogeneous, but the age range in each session was similar, ie., women of similar ages were not clustered in specific sessions. Men were not clustered into separate groups from women. The extent of dry eye symptoms (based on a questionnaire) and objective clinical signs (corneal fluorescein dye staining, Schirmer test, tear break up times) of the dry eye people selected from the dry eye clinic in SNEC have been reported in other papers [42].

## Results

**Fig. 1:**
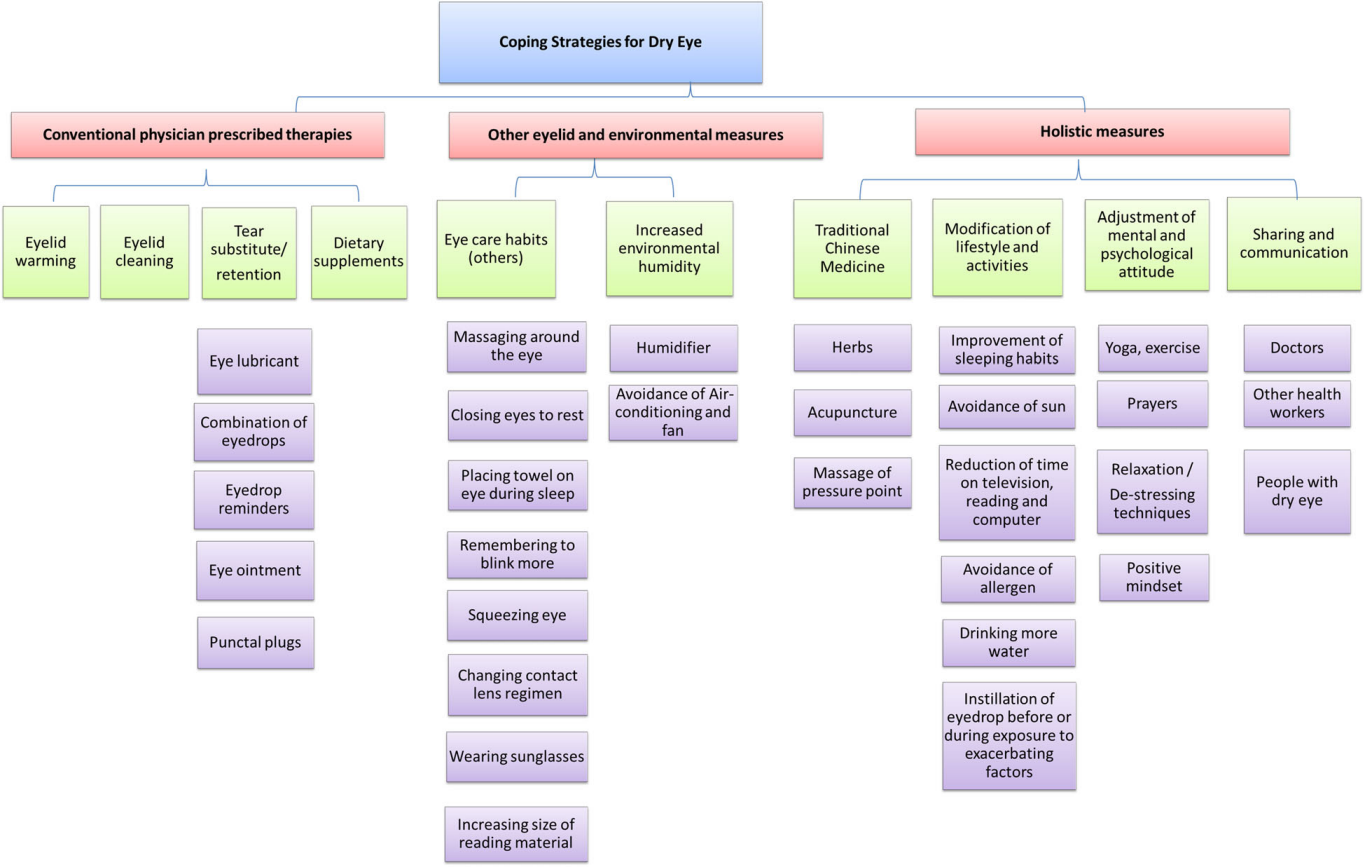
Schematic illustrating the coping strategies uncovered in focus groups

The various themes identified from the data were described below and summarised in Fig. 1. The primary themes were: (a) eyelid warming, (b) eyelid cleaning, (c) tear substitute/ retention, (d) dietary supplements, (e) modification of eye-care habits other than warming or cleaning, (f) modifying environmental humidity, (g) traditional chinese medicine (TCM), (h) modification of lifestyle and activities (eg. Sleeping habits, wearing sunglasses), (i) adjustment of mental and psychological attitude, and lastly (j) sharing and communication related to dry eye. The first 4 themes were commonly associated with counselling received from eye-care professionals, whereas the other 6 themes could be uncommonly discussed by professionals. Examples of the strategies in their respective themes are shown in Additional file 1: Table S1 while number of times each theme was mentioned can be found in the Additional file 1: Table S2.

### Eyelid warming

A big proportion of participants commented that regular warm compress has helped them to self-manage the ‘dry eye condition’. Different methods of warm compress such as the use of electrically powered steaming goggles, eye masks and hot towels were mentioned. Warm compress has also allowed a participant to see clearer: “when I put the warm compress, I see clearer. Sometimes, when I instill the eye drop, I cannot see. The vision is still as bad. After using the warm towel, I can see better”. Another commented, “When I use the eye mask, the oil glands get emptied and I feel better because of that”. Another individual noted that warm compresses with the eye mask are ‘very relaxing’ and she will normally perform this on her closed eyes for 10 min without fail. Patients who are registered patients at the SNEC’s dry eye clinic have been advised to perform eyelid warming at least 8 min a day.

### Eyelid cleaning

The use of eyelid cleaning agents was reported to be especially beneficial for participants who wear eye-related make-up. One lady commented: “Another issue is wearing cosmetic, as it might not get removed effectively. I use Blephagel to clean the lids, especially when eyeliner has been used, Blephagel helps to clean it off… as a result I don’t feel so uncomfortable and also use less eye drops”. Another participant shared, “The gel makes your eyesight blur but when applied before going to bed, it is fine. It will be sticky the next morning so I use a special cleanser to clean”. Participants tend to feel that eyelid cleaning is a useful additional measure to ocular lubricants to control dry eye. A minority of participants found eyelid cleaning to be inconvenient and do not help with the dry eye symptoms.

### Tear substitute/retention

The most commonly used measure for dry eye is tear substitute and in Singapore it is readily available over-the-counter in different formulations such as lubricants and ointments. Among the self-management methods, this tends to be most frequently mentioned, and one distinctive theme is that different formulations are preferred by participants. Some participants commented that the combinatorial use of oil-based and aqueous based lubricants may help, and the use of oil-containing lubricants followed 10 min later with water based drops increased the symptomatic relief. Methods of reminding oneself to instil eye drops (using an alarm, placing lubricants where it is prominent) have also been mentioned.

One lady noted, “One just have to keep applying eye drops… so many times. That’s the only way to get relief”. From the conversations, it seems that dry eye sufferers are not satisfied with eye lubricants if they are used solely. This is partly due to the requirement for frequent instillation. For participants who mentioned only single coping methods, those in this category appeared to be least satisfied.

Punctal plugs are normally inserted by medical practitioners to reduce the rate of tear outflow through the lacrimal canaliculi, so this is not a self-management measure. This is favoured by some participants, and though not strictly an intervention of the physical environment, it is included in this category as it is an intervention on the ‘moisture level’ of the ocular surface.

### Dietary supplements

A few participants reported that omega-3 supplements have helped to reduce the frequency and severity of their dry eye symptoms. A participant commented, “I also take omega 3, I find that there is a relief”. However, participants who took omega-3 supplements also adopted other treatment modalities such as tear substitutes and other measures with modification to their activities and lifestyles as described below. There was no predisposition for participants of any socio-economic group for this form of remedy.

### Modification of eye-care habits other than warming or cleaning

Methods such as massaging around the eye, closing and resting the eye, placing towel on eye during sleep, remembering to blink more frequently, and rubbing/squeezing the eye have been reported to provide symptomatic relief by some participants. Changing the usage of contact lens and wearing sunglasses were also used by participants. Wearing sunglasses help to prevent photophobia associated with dry eye and increasing the size of reading fonts to facilitate ease of reading were also adaptive measures to cope with dry eye induced blurring of vision.

### Modifying environmental humidity

Humidity levels have been reported by participants to affect the frequency and severity of dry eye symptoms. Methods to modify the humidity of the living environment were discussed during the focus groups, and these include the use of humidifier and avoidance of air-conditioning and fans. One participant commented, “I can sleep better with a humidifier and feel that my eyes, skin and throat are less dry”. Another man stated, “Once, I experienced dry eye when I was driving, and I felt that my eyes get irritated and very dry when the air conditioner is blowing on my face. After that incident, I tried to drive less often”. Other adults also mentioned that they would try to avoid air-conditioned places; they would prefer a place with no air-conditioning and fan. Adjusting the draft from air conditioners and fans was an adaptive measure shared by a few participants. A lady noted, “My grittiness comes now and then. I cannot stay in the air con room for long. I cannot stand the air con, have to lower the fan or adjust the direction. The fan has to be at a certain angle, cannot face me”.

### Traditional Chinese medicine

TCM is the most common form of complementary medicine practiced in Singapore, with increasing popularity. This includes the oral consumption of herbs, acupuncture treatment and pressure over specific body points. One individual commented, “tried (LingZhi) Chinese herbs that help to improve sleep over a period of time, it helps with dry eye as well”. In this particular case, the Chinese herbs help the participants to have a better quality of sleep and obtaining relief from her dry eye symptoms indirectly. Another participant mentioned, “they treat using acupuncture. Mine is dry eye. So it helps. After it was taken out, you can see very clearly, my condition was dry eye, quite bad already, so I have to keep on going there, every week. There was this TCM practitioner, he is very good, immediately able to see me, he said I have dry eye. He also said to try acupuncture. Immediately after the treatment, so clear, the words all appear to be very clear. Only acupuncture was done, nothing else”. Other participants indicated that acupuncture treatment will generally take a few sessions over a longer period of time to produce sustained improvement. Many participants were eager to learn more from those who benefited from pressure point massage as well.

### Modification of lifestyle and activities

Modification of lifestyle has been commonly reported during the focus groups. Having sufficient good quality sleep helps many participants. One participant commented, “My personal experience is if I have more than 8 hours of sleep, I am fantastic. My eye will not be dry, but will be brighter and bigger. If I don’t have sufficient sleep, my eyes will be somewhat like now: small and dry”. Reduction of the palpebral fissures due to a protective response is commonly referred to as having ‘small eyes’ in the local context. Another lady stated, “About one year ago, I tell myself to sleep more, sleep more and sleep more. And from then it actually improved my dry eye, the discomfort level is not as severe as before”. Although many of the participants agreed that having sufficient and good quality sleep are associated with significant symptomatic improvement in dry eye, others emphasized that total psychological health is important to overcome dry eye condition.

Avoidance or reduction of certain activities including outdoor exposure to sun, television watching, reading and computer usage was noted. Increasing the frequency of instillation of lubricants was a common behavioural adaptation when exposed to exacerbating factors. One man noted, “In the movie, I will lubricate before the movie, half way through and at the end of the movie”. Another participant commented, “In the plane I use more (eyedrops). In winter and autumn climates, I apply more (eyedrops)”. Avoidance of allergens and dehydration by drinking plenty of water has also been mentioned. One participant with Sjogren syndrome, currently teaching in a school, shared, “it can be itchy due to the dust (in the classroom). The ideal situation is, if the place is known to be dusty, use eye drops before entering, at least 15 minutes (before), let it ‘absorb’ first. That is my own experience”. Overall the participants agreed that modifying lifestyles do help to relieve dry eye.

### Adjustment of mental and psychological attitude

The power of thoughts and emotions has shown to influence the dry eye condition in many individuals, as indicated by one participant, “I sleep well and it is the mental, positive thinking, telling myself that I can manage all this. I discover this power”. Another lady shared, “I do my prayer. I always tell God, on the strength of Jesus, I am healed. Every day I pray in this positive way”. Other than having a thankful and optimistic mind set, many adults also reported that relaxation and de-stress techniques have helped to effectively manage dry eye, measures include the practice of yoga for eye, qi-gong, running, other aerobic exercise, being more happy in general, spending time with friends and family, laughing out at things, doing things for others, eating healthily, playing games that one enjoys. One 58 year old part-time teacher with Sjogren syndrome commented, “If I am not so stressed, my eye is fantastic… I know what triggers my symptoms…And I will keep my mind (occupied), I try not to occupy myself by going to the shopping centre, this will encourage spending. Instead, I will take the bus from one bus interchange to another bus interchange, and back again… I will sing songs during the journey. That is how I release stress…”This participant indicated that when she is stressed, other symptoms of Sjogrens such as dry mouth and swollen lymph nodes will also emerge. Many participants had to change jobs as a result of dry eye, “I need to save costs because I cannot find a full time job… but I will do unpaid volunteer work to occupy my mind and to overcome the stress. I will not be alone, tell myself, never ever be alone or it will easily cause me to be stressed”.

When asked about how to relieve stress, one lady said, “Nothing much, I will walk every morning without fail and do my exercise for about one and half hour. I stay near a park so it is easy to run in a loop. I am happy doing my housework. I have a daughter and she is already married so I feel quite lonely in the house when she goes to work. Doing housework and cooking do help. Some of my sisters tell me to stop cooking for my daughter now that she is married, I said: In that case I have nothing to do and I am happy doing that (cooking)”. Another participant commented, “I love dogs and I like to rescue dogs. I also spend time with my grandchildren, so all the pain did not get to me as much when I am with dogs (at a shelter). I am very concerned about them, when I am with my grandchildren I am also very concerned about them. The pain in the eye does not bother me so much then. I really love dogs and being with them, and then I do not get bothered with my eyes”. It is evident that participants who engaged meaningfully in activities that they enjoy have a higher level of happiness and these also help to divert attention from the dry eye symptoms.

### Sharing and communication related to dry eye

A few participants noted that counselling on dry eye help them to manage their condition better. One individual commented that it helped to be thoroughly evaluated and counselled on each visit, “someone in the clinic to talk to every visit, to be evaluated with forms and to be asked if the eye drops are helping or otherwise, it helps to know if different kinds of eye drops can be used for different purposes”. Other participants reported that it is reassuring to talk to health-care professionals, but understanding more about the condition will also improve their ability to self-manage.

## Discussion

The coping strategies identified in this paper provide useful insight on how people from different backgrounds and situations cope with dry eye. Some methods such as tear substitute and warm compresses are common strategies mentioned. Different tear lubricant formulations are preferred by individuals, this is consistent with the findings that patients prefer eye drops with a range of osmolarities and acidities [46]. It needs to be emphasized that both tear substitutes and warm compresses are long-term treatments and most participants are reliant on them for relief. This may be due to the high prevalence of meibomian gland dysfunction in the people with dry eye.

Using a combination of coping strategies (eyedrops, humidifiers, avoiding of direct fans) have also been frequently reported during the focus groups as being effective. For instance, placing a humidifier next to the bed and redirecting the fan to avoid windy drafts on the face. Each individual seems to have a unique preference and combination of measures to manage dry eye, perhaps related to differences in circumstances as well as personalities. For some patients, the measures have arisen from detailed discussion with a physician or health care professional, which may over many frequent visits, while other participants have discovered the optimal strategies largely on their own effort/experimentation. Since participants are unlikely to use single modalities such as oral health supplements alone, one cannot interpret their efficacy or usefulness. Whilst participants may be enthusiastic about using acupuncture, some of the immediate benefits are likely due to reflex tearing and may not have long term effects.

A holistic approach seems to be essential and under-rated in the treatment of dry eye condition. The role of qigong and outdoor exercises for example, has not been evaluated in the peer reviewed literature in dry eye. Participants appeared to be most excited about holistic measures, rather than well-known measures such as eye drops. This may be partly attributed to a desire to educate others. Perhaps such an attitude can be used for the purpose of systemic education by counsellors in dry eye. Patients appear to appreciate systemic step by step approach to dry eye.

In the research on other chronic diseases, leisure has been proven to be ‘therapeutic’, and has the ability to improve general well-being [47]. It should be highlighted that participants reported that doing enjoyable things and being passionate about things help in the management of dry eye. Some participants have found distraction through an unrelated activity to divert focus from the discomfort associated with dry eye. It is not clear if a totally positive engagement with life or an approach based on distraction is more effective. Either way, being passive appears to be not beneficial and in fact, may be predisposing for depression [48–52].

One limitation of this study is that most of the participants were female and attitudes towards disease may be gender dependent, so limiting the ability to extrapolate to all dry eye sufferers. Nevertheless the majority of people with dry eye in the population may also be female [53]. Focus groups were the only source of data for this report, and this method of data collection has some limitations. This approach does not accurately document the relative frequency of strategies used, and unless stratified into separate studies/groups, cannot compare the relative use of the strategies in people with different severities of dry eye. Findings may not truly represent views from all participants. Participants who had been more vocal had a tendency to dominate the focus groups, and possibly suppress comments and views of other individuals. It is possible that some men and younger women may not be completely honest in making their views in the presence of older women, though the facilitator did not notice such a phenomenon. Efforts were made by the facilitator to get equal participation from all. Even though a few sessions have been held, samples are still small and could be biased. In particular, patients who were unhappy with their clinical management for any reason, could have refused to participate in the focus groups. Lastly, as with other qualitative methods, bias and subjectivity of interpretation may not be avoidable, despite having 3 assessors.

## Conclusion

In conclusion, we found holistic coping strategies to be prominent in dry eye sufferers from these focus groups, and more qualitative medicine approaches may need to define the targeted treatment outcomes in dry eye. Clearly an approach based solely on severity/regularity of dry eye symptoms and signs will be unlikely to represent the true burden of dry eye in society.

## Additional file

Additional file 1: Supplementary materials. Script for Dry Eye Focus Group. **Table S1**. Examples of strategies used in the themes uncovered in the focus groups. **Table S2**. The number of times focus group themes were mentioned by participants. (DOCX 21 kb)

## Abbreviations

DED: Dry eye disease
QoL: Quality of life
SNEC: Singapore National Eye Centre
TCM: Traditional Chinese Medicine
