# Supplementary material for: Coping with dry eyes: a qualitative approach
Source: BMC Ophthalmol. 2018 Jan 16;18:8. doi: 10.1186/s12886-018-0671-z (PMC5771005; doi:10.1186/s12886-018-0671-z)
Supplement: Supplementary file 1 — Supplementary materials. Script for Dry Eye Focus Group. Table S1. Examples of strategies used in the themes uncovered in the focus groups. Table S2. The number of times focus group themes were mentioned by participants. (DOCX 21 kb) [file 12886_2018_671_MOESM1_ESM.docx]

**Supplementary Materials**

Script for Dry Eye Focus Group

- What strategies were adopted to cope with your dry eye conditions?
- Are different strategies used under exacerbating factors (based on what was shared)?
- How do you perform the mentioned technique/method?
- What do you do when the dry eye symptoms are bad?
- Can you describe on the mentioned technique/method that you think have helped in your dry eye self-management.
- How do you think this method has helped in your dry eye self-management?
- Tell us more about the method you mentioned previously.
- Are there any other things you do that can help to alleviate the dry eye symptoms?
- What do you think have helped to relieve your dry eye symptoms?
- What do you think have made you able to self-manage your dry eye conditions?
- Can you describe any non-conventional methods that you think have helped in your dry eye management?
- What are the changes made to your daily lifestyle that has helped you in coping with the dry eye condition?
- What do you think have helped you the most?
- How does the mentioned technique alleviate your dry eye symptoms?
- What have you been doing to take care of your eyes?
- What are the different strategies used at different time of the day?
- What are the specific steps for the mentioned coping strategies?
- What are the alternative methods that you have tried to relieve dry eye symptoms?
- Which treatment helped you the most?
- What are the impacts of your dry eye in daily activities, how do you manage them?
- Is there anything to add on the mentioned strategies?
- What are the modalities that you have tried that relieves dry eye symptoms?
- Other than the mentioned strategies, what are the other methods that you have adopted?
- How often do you do the mentioned treatment?
- What is the specified type of mentioned treatment that you used?
- What other products do you use that you think has helped in your self-management strategies?
- Are there any other strategies that you used to cope with your dry eyes?
- How do you cope with your dry eyes?
- Is there any strategies adopted that you find especially useful to prevent the dry eye symptoms from flaring up?
- Is there any strategy used that is different from daily regime when you are doing something or going to a place that you know will make your dry eye symptoms worse?
- Are there any methods that help you to remember your dry eye regimen every day?

**List of supplementary tables:**

**Supplementary Table 1.** Examples of strategies used in the themes uncovered in the focus groups

**Supplementary Table 2.** The number of times focus group themes were mentioned by participants

**Supplementary Table 1.** Examples of strategies used in the themes uncovered in the focus groups

| **Strategies** | **Examples** |
| --- | --- |
| **Conventional physician prescribed therapies** | |
| **Eyelid warming** | "use the hot pad" "do warm compress" |
| **Eyelid cleaning** | "clean my eyes with blephagel" |
| **Tear substitute/ retention** | "use Tear Naturale" "use combination of Thera tear  and Endura eye drops" |
| **Dietary supplements** | "had Thera tears capsule" |
| **Other eyelid and environmental measures** | |
| **Eyecare habits (others)** | "massage around my eye" "close my eyes to rest" "remind myself to blink more" "stopped wearing contact lenses" |
| **Increased environmental humidity** | "put humidifier next to my bed" |
| **Holistic measures** | |
| **Traditional Chinese Medicine** | "Acupuncture helps" |
| **Modification of lifestyle and activities** | "sleep more" "avoid dusty places" |
| **Adjustment of mental and psychological attitude** | ""try to relax" "positive thinking" |
| **Sharing and communication** | "hearing from the specialist" "talking to people with the same sickness" |

**Supplementary Table 2.** The number of times focus group themes were mentioned by participants

| **Coping Strategies for Dry Eye** | |
| --- | --- |
| **Themes** | **Number of times mentioned** |
| **Conventional physician prescribed therapies** |  |
| **Eyelid warming** | 23 |
| **Eyelid cleaning** | 17 |
| **Tear substitute/ retention** |  |
| Eye lubricant | 48 |
| Combination of eyedrops | 1 |
| Eyedrop reminders | 5 |
| Eye lubricant | 10 |
| Punctal plugs | 2 |
| **Dietary supplements** | 7 |
| **Other eyelid and environmental measures** |  |
| **Eyecare habits (others)** |  |
| Massaging around the eye | 3 |
| Closing eyes to rest | 1 |
| Placing towel on eye during sleep | 1 |
| Remembering to blink more | 3 |
| Squeezing eye | 1 |
| Changing contact lens regimen | 1 |
| Wearing sunglasses | 4 |
| Increasing size of reading material | 1 |
| **Increased environmental humidity** |  |
| Humidifier | 4 |
| Avoidance of Air-conditioning and fan | 5 |
| **Holistic measures** |  |
| **Traditional Chinese Medicine** |  |
| Herbs | 1 |
| Acupuncture | 1 |
| Massage of pressure point | 2 |
| **Modification of lifestyle and activities** |  |
| Improvement of sleeping habits | 8 |
| Avoidance of sun | 1 |
| Reduction of time on television, reading and computer | 8 |
| Avoidance of allergen | 2 |
| Drinking more water | 1 |
| Instillation of eyedrop before or during exposure to exaberating factors | 5 |
| **Adjustment of mental and psychological attitude** |  |
| Yoga, exercise | 2 |
| Prayers | 1 |
| Relaxation/De-stressing techniques | 18 |
| Positive mindset | 3 |
| **Sharing and communication** |  |
| Doctors | 3 |
| Other health workers | 1 |
| People with dry eye | 1 |
